# A Study of* Trichomonas vaginalis* Infection and Correlates in Women with Vaginal Discharge Referred at Fann Teaching Hospital in Senegal

**DOI:** 10.1155/2019/2069672

**Published:** 2019-04-01

**Authors:** Roger C. Tine, Khadime Sylla, Rougyatou Ka, Lamine Dia, Doudou Sow, Souleye Lelo, Khardiata Diallo, Babacar Faye, Thérèse Dieng, Cheikh T. Ndour, Ahmet Y. Sow

**Affiliations:** ^1^Department of Medical Parasitology, Faculty of Medicine, University Cheikh Anta Diop, Dakar, Senegal; ^2^Laboratory of Parasitology and Mycology, Fann Teaching Hospital, Dakar, Senegal; ^3^Laboratory of Bacteriology and Virology, Fann Teaching Hospital, Dakar, Senegal; ^4^Department of Infectious and Tropical Diseases, Fann Teaching hospital, Dakar, Senegal

## Abstract

**Introduction:**

Trichomoniasis is nowadays the most prevalent non-viral sexually transmitted infection in the world. In Senegal, the epidemiology of trichomoniasis is not well known. The current study aimed at assessing the prevalence and factors associated with* T. vaginalis* infection among women with vaginal discharge.

**Methods:**

A retrospective analysis of laboratory records from patients referred at the Fann Teaching Hospital in Dakar, Senegal, for vaginal discharge was carried out. The study covered the period from 2006 to 2011. For each participating woman, a vaginal swab was collected and a wet mount smear performed immediately. Optic microscopic examination with 40x magnification was done to detect* T. vaginalis* and assess biological modifications such as presence of epithelial cells, white blood cells, and red blood cells. A gram stained smear was also performed and examined under oil immersion (100x magnification) to assess the vaginal flora.

**Results:**

Overall, 3893 women were enrolled with a mean age at 31.2 ± 10 years. The prevalence of Trichomoniasis represented 4.8%, 95%CI(3.1-5.7) and it was lower among women less than 30 years (4.1%), while divorced women more likely to be infected compared to married and single women (aOR:2.1, 95%CI (1.2-3.7)). Trichomoniasis was associated with abnormal vaginal flora such as type III (aOR:2.6, 95%CI(1.5-4.4)) and type IV (aOR:3.3, 95%CI(2.1-5.3)). In addition, patients with erythrocytes excretion were more likely to be infected by* T. vaginalis* (aOR:2.8, 95%CI(1.9-3.9).

**Conclusion:**

* Trichomonas vaginalis *remains prevalent among sexually active women. Strategies aiming at improving disease awareness in these high-risk groups are needed to improve trichomoniasis prevention but extensive epidemiological data are still needed for a better understanding of the disease transmission dynamic.

## 1. Introduction

Trichomoniasis is the most prevalent non-viral sexually transmitted infection in the world [1].* Trichomonas vaginalis*, the causative agent is a protozoan parasite infecting the urogenital tract of both females and males [2]. It is reported to be 250 million new cases worldwide every year [3] and Trichomoniasis accounts to almost half of curable sexually transmitted infections according to the world health organisation [3, 4]. In general, the infection is asymptomatic in men although it can be associated with urethral discharge and dysuria [5], while infected women can have different symptoms consisting in yellowish-green frothy discharge, purities, dysuria, and the strawberry cervix which is recognized by punctuates haemorrhagic lesions [5]. Infection by* Trichomonas vaginalis* among women can lead to serious complications such as adverse pregnancy outcomes that appear by preterm rupture of membranes, preterm delivery, low birth-weight infants, infertility, and cervical cancer [6]. Moreover, studies have shown an increased risk of HIV transmission among individuals infected by* T. vaginalis* [7].* Trichomonas vaginalis* transmission is very heterogeneous and depends on several factors; it is established that socioeconomic status, age, hygiene habits, sexual behaviour, phase of the menstrual cycle, and other concomitant sexually transmitted infection can play a key role on the disease burden [8, 9]. The prevalence and the average duration of Trichomonas infection mainly depend on the health care seeking behaviour of population and their access to health care [10]. Primary prevention of* Trichomonas vaginalis* infection often relies on health promotion interventions to improve diseases awareness and behaviour change [11]; but male circumcision represents an important means for the prevention of* T. vaginalis* transmission and several studies have shown that partners of circumcised men are less at risk of acquiring sexually transmitted infections including Trichomoniasis [12, 13]. Oral metronidazole remains the recommended drug regimen for the treatment of trichomoniasis and concurrent treatment of sexual partners is recommended to prevent reinfections [14]. 

In many settings including Senegal, patients presenting at primary care units with signs suggestive of STI (urethral discharge, vaginal discharge syndromes) are often being diagnosed and managed presumptively using a syndromic approach based on WHO guidelines [15]. But studies have shown that a syndromic-based approach in some settings may lack sensitivity and specificity and can lead to mismanagement of several STI including trichomoniasis [16, 17]. In addition, biological confirmation of* T. vaginalis* infection in many primary care units remained at a low level due to lack of appropriate diagnostic tool and community prevalence data remained scarce [18, 19]. Thus, limited data regarding the epidemiology of Trichomoniasis are available especially among at risk population such as women of reproductive age. A better understanding in the epidemiology of* T. vaginalis* is thus needed and may help shape existing control strategies and treatment practices regarding STI in Senegal. To overcome these gaps, this six-year trends analysis was conducted to provide insight into the prevalence of* T. vaginalis* infection among women with vaginal discharge referred at the Fann teaching hospital in Dakar, Senegal, and explore potential factors associated with* T. vaginalis* infection.

## 2. Methods

### 2.1. Study Settings

The study was conducted at the Fann teaching hospital, which is a public referral hospital, located in the capital city of Dakar. Population access to this referral hospital including access to laboratory services is easy by simple appointment. Although data on trichomoniasis in Senegal has scarcely been described, prevalence of STI in the general population is still low [20, 21] and their management under routine conditions usually refers to a syndromic based-approached [16].

### 2.2. Design and Population

The present study is a retrospective analysis of data from patients referred at the Fann teaching hospital for vaginal discharge during the period from 2006 to 2011. Participating women were eligible if they had at least 18 years. Women, who were previously screened for STI within the same study period, were excluded in the analysis. A code was given to each enrolled participants and data on women's sociodemographic characteristics and residency were collected from participant's medical records based on prior permission from the administration officials of the Fann Teaching Hospital.

### 2.3. Specimen Collection and Processing

For each participating woman, a vaginal swab was collected and a wet mount smear was performed immediately as part of a routine diagnostic procedure for a motile parasite. The wet mount smear was examined using optical microscopy with 40x magnification to detect* T. vaginalis* and assess biological modifications such as presence of epithelial cells, white blood cells, and red blood cells.* Trichomonas vaginalis *infection was considered on the basis of a positive result from a wet mount microscopy of motile trichomonad. The magnitude of white and red blood cell within the vaginal discharge was classified as follows: (i) rare 1 to 5 cells/field microscopy, (ii) moderate: 6 to 10 cells/field microscopy, (iii) many: 11 to 20 cells/field microscopy, and (iv) high: 21 cells and above/field microscopy. In addition, a gram stained smear was performed to characterise the vaginal flora using Nugent scoring [22]. Briefly, each Gram-stained smear was evaluated for the following morphotypes under oil immersion (100x magnification): large gram-positive rods (lactobacillus morphotypes), small gram-variable rods (*G. vaginalis* morphotypes), small gram-negative rods (Bacteroides spp. morphotypes), curved gram-variable rods (*Mobiluncus spp*. morphotypes). Each morphotype was quantitated from 1 to 4+ with regard to the number of morphotypes per oil immersion field and the vaginal flora was characterised as follows: Type I: less than 1 morphotype; Type II: 1 to 4 morphotypes; Type III: 5 to 30 morphotypes; Type IV: 30 or more morphotypes) as described elsewhere [22]. Types I and II were considered as normal vaginal flora, while types III and IV were considered as abnormal flora.

### 2.4. Statistical Methods

Data were entered into Filemaker Pro™ software and extracted for cleaning and analysis using STATA software (version 14.0 - StataCorp LP, Texas). For binary data, percentage was used to assess the frequency of each outcome with a 95% confidence interval (95%CI). For continuous data, mean and standard deviation were used to describe normally distributed variables. Characteristics of all women included in the study were tabulated. Proportions were compared using chi square test (univariate analysis). Prevalence of* T. vaginalis *was calculated and expressed as proportion with 95%CI. To assess factors associated with* T. vaginalis* infection a multivariate logistic regression with adjustment on covariates such as age group, study period, and marital status was done. From the final model, adjusted odds ratios were derived with their 95%CI. Model validity was tested using the Hosmer-Lemeshow goodness-of-fit test. The performance of the final model was assessed by the area under the curve and Akaike and Bayesian information criterion; in addition, a test for multicolinearity between variables was done using the variance inflation factor. Significance level of the different tests was 0.05, two-sided.

## 3. Results

### 3.1. Participant's Characteristics at Enrolment

Overall, for the six-year period, 3893 women with vaginal discharge were referred at the Fann Teaching Hospital for aetiological investigation. The mean age of the study participants was 31.2 ± 10 years and the majority were below the age of 35 years. Indeed, 25.9% of the women were less than 25 years old and 30.7% of them had an age ranging from 25 to 35 years. Married women represented a proportion of 53.8%, while single and divorced women represented, respectively, 20.1% and 6.5%.  Table 1 summarises study participants characteristics at enrolment.

### 3.2. Microscopic Findings among the Study Participants

Optic microscopic examination of wet mount smear revealed the presence of white blood cells among 3789 participants (97.3%). Presence of white blood cell within the vaginal discharge was categorised as rare for 2947 participants (75.7%), moderate for 388 participants (9.9%), many for 242 participants (6.2%), and high for 212 participants (5.4%).

Out of the 3893 examined vaginal swabs, 561 (14.4%), were found with red blood cells. The importance of these red blood cells was considered as rare for 169 specimens (4.3%), moderate for 285 specimens (7.3%), many for 82 specimens (2.1%), and high for 25 specimens (0.6%). Giemsa stained smear microscopic examination revealed that 1186 women had a type I vaginal flora (30.5%), while 634 (16.3%) had a type II vaginal flora. Abnormal vaginal flora type III and type IV represented, respectively, a proportion of 14.8% and 38.4% (Table 2).

### 3.3. Prevalence and Trends of* Trichomonas vaginalis*

Among the 3893 patients referred at the teaching hospital for vaginal discharge, 189 of them were infected by* Trichomonas vaginalis* providing a prevalence of 4.8%, 95%CI (3.1 - 5.7).

Analysis of* T. vaginalis* distribution by age group revealed increasing prevalence as age group increases. Among women less than 25 years old, a prevalence of 4.3% was noted, while it was at 4.1% among women with an age between 25 to 30 years old.* T. vaginalis* prevalence was at 5.2%, 6.5%, 6.1%, and 5.2%, respectively, among women with an age range between 31 to 35 years, 36 to 40 years, 41 to 45 years, and more than 45 years. The highest prevalence was noted among divorced women (11.1%) followed by non-married women (single) (4.3%) and married women (3.9%). In 2006, a prevalence of 6.7% was noted versus 10.8% in 2007. Among the 433 examined participants in 2008, 28 of them were found with* T. vaginalis* providing a prevalence of 6.5%.* T. vaginalis* prevalence was at 4.1%, 3.2%, and 3.4%, respectively, in 2009, 2010, and 2011 (Table 3).

In a multivariate logistic regression analysis, after adjustment on covariate such as study period, age group, and other biological changes, divorced women were more likely to be infected by* T. vaginalis* compared to married and single women (adjusted OR: 2.1, 95%CI: (1.2 - 3.7) (Table 5).

### 3.4. Relationship between Biological Changes and Frequency of* T. vaginalis*

Among the examined vaginal swabs for which no leukocytes were found,* T. vaginalis* was identified with a proportion of 2.9%, while that was at 3.5% for specimen with 1 to 5 leukocytes/field microscopy. Frequency of* T. vaginalis* was, respectively, at 8.8%, 8.7%, and 13.7% for specimen with moderate, many, and high white blood cells excretion.* T. vaginalis* was isolated with a frequency of 3.9% within the vaginal swab where no erythrocyte was identified; the proportion of vaginal swabs with rare excretion of erythrocytes for which* T. vaginalis* was identified represented 11.8%. Among samples with moderate, many, and high excretion of erythrocytes,* T. vaginalis* was found with a proportion of 8.8%, 13.4%, and 8.0%, respectively.

Frequency of* T. vaginalis* among participants with a vaginal flora classified as type I was at 2.0% versus 3.1% for patients with type II vaginal flora. For participants with type III vaginal flora,* T. vaginalis* was identified with a frequency of 5.7%; for patients with type IV vaginal flora, the parasite was present with a frequency of 7.5% (Table 4).

In a multivariate logistic regression analysis, presence of* T. vaginalis* was significantly associated with vaginal flora; patients with abnormal vaginal flora such as type III (adjusted OR: 2.6) and type IV (adjusted OR: 3.3) were more likely to be infected by* T. vaginalis* compared to those with normal vaginal flora. The presence of erythrocytes within the vaginal swab increased the likelihood of* T. vaginalis* infection by 2.8-fold (Table 5).

## 4. Discussion

*Trichomonas vaginalis* is one of the most common STI in the world but its prevalence is very heterogeneous across countries [23, 24]. In Senegal, data on* T. vaginalis *epidemiological profile remains limited. The current study was conducted to describe the prevalence of* T. vaginalis* among women with vaginal discharge and explore potential factors associated with trichomonas infection. The study revealed an overall prevalence of* T. vaginalis* of 4.8% but the disease distribution across age groups remained heterogeneous; women with an age range between 31 to 45 years were the most infected population (prevalence range: 5.2 % - 6.5%). These findings are consistent with data from other studies that showed that 25- to 45-year-old women are at higher risk of being infected by* T. vaginalis* [25–27]. Trichomoniasis in that age group is more prevalent due to the fact that it is a sexually active and reproductive age group, which is predisposing factor for infection [9, 28]. Thus, strategies aiming at improving disease awareness in this high-risk group are needed to further improve trichomoniasis prevention.

The frequency of* T. vaginalis* infection in this study was lower than what was reported in other African settings such as Zimbabwe where a prevalence of 9.5% was found [29], but it is consistent with the reported prevalence of 5% in the Pakistan region [30]. In contrast, higher prevalence was reported in the USA among imprisoned women [31]. These variations can be due to variability in terms of disease exposure [9] as well as use of different diagnostic methods across studies. Unlike other studies that showed higher prevalence among married women [26, 32], this study revealed that divorced women were more likely to develop trichomoniasis compared to single and married women. However, without additional information on participant's sexual behaviour, educational level, or knowledge about STI, it was not possible to clearly explain the relationship between marital status and trichomoniasis prevalence. Epidemiological studies have established that low educational level, smoking, and sexual behaviours are significantly associated with* Trichomonas vaginalis* infection [8, 9] but our study did not collect information on these variables.

The data showed significant biological modifications associated with* Trichomonas vaginalis* infection. Indeed, patients with red blood cell within the vaginal swabs were more likely to be infected compared to patients for whom no red blood cell was found in the vaginal swab. Infection with* T. vaginalis* can result in significant inflammatory and cytolytic actions induced by the parasites itself and the severity of these pathogenic actions depends on the host and* T. vaginalis* strains [33, 34]. Moreover,* T. vaginalis* infection was associated with significant modification of the vaginal flora and participants with abnormal vaginal flora (Types III and IV) were more likely to be infected. These findings are consistent with data from longitudinal studies that have shown increasing risk of acquiring* T. vaginalis* among patients with bacterial vaginosis [35, 36] as it was the case in our study.

The study has some limitations. In the current study,* Trichomonas vaginalis* detection was only based on wet mount smear microscopic examination as part of a routine standard practice and no additional investigations such as culture or PCR were done. This may have lowered parasite detection rate. It is well established that, among the available methods, at least two methods are better for diagnosis of* T. vaginalis*, for example, culture and wet mount microscopy [32]. However, culture is often subject to contamination by bacteria that may hinder its growth [37, 38] and in the study a higher prevalence of T. vaginalis was noted among patients with bacterial vaginosis.

## 5. Conclusion

*Trichomonas vaginalis *remains prevalent among women with vaginal discharge with a higher burden among sexually active women. Strategies aiming at improving disease awareness among these high-risk groups are needed and should include health promotion, education, and prevention with regard to sexual behaviour. However, extensive epidemiological data are needed to better understand the epidemiology of* T. vaginalis*.

## Figures and Tables

**Table 1:** Study participants' characteristics.

Parameters	Number	Frequency	95%CI

*Age group*∗			
Less than 25 years	1008	25.9	24.5 - 27.3
25 to 30 years	1194	30.7	29.2 - 32.1
31 to 35 years	613	15.7	14.6 - 16.9
36 to 40 years	443	11.4	10.4 - 12.4
41 to 45 years	310	7.9	7.1 - 8.8
46 and more	325	8.3	7.5 - 9.3

*Marital status*			

Single	782	20.1	18.8 - 21.4
Divorced	252	6.5	5.7 - 7.3
Married-monogamous	2095	53.8	52.2 - 55.4
Married-polygamous	764	19.6	18.4 - 20.9

*Study period*			

2006	832	21.4	20.1 - 22.7
2007	148	3.8	3.2 - 4.4
2008	433	11.1	10.1 - 12.1
2009	994	25.5	24.2 - 26.9
2010	1075	27.6	26.2 - 29.0
2011	411	10.6	9.6 - 11.6

*Total number of participants*	*3893*		

∗Mean age: 31.2 ± 10 years.

**Table 2:** Microscopic findings among participating women.

	Number	Frequency (%)	95%CI

*Leukocytes (WBC)*			
Absence	104	2.7	2.2 - 3.2
Rare	2947	75.7	74.3 - 77.0
Moderate	388	9.9	9.0 - 10.9
Many	242	6.2	5.5 - 7.0
High	212	5.4	4.7 - 6.2

*Red Blood cell (Erythrocytes)*			

Absence	3332	85.6	84.4 - 86.7
Rare	169	4.3	3.7 - 5.0
Moderate	285	7.3	6.5 - 8.2
Many	82	2.1	1.7 - 2.6
High	25	0.6	0.4 - 0.9

*Vaginal flora*			

Type I	1186	30.5	29.0 - 31.9
Type II	634	16.3	15.1 - 17.5
Type III	576	14.8	13.7 - 15.9
Type IV	1497	38.4	36.9 - 40.0

∗Assessed by Nugent Score.

**Table 3:** Prevalence and distribution of *Trichomonas vaginalis* infection among the study participants.

Parameters	Number of examined women	Number of positive	Prevalence (%) (95%CI)

*Overall*	3893	189	4.8 (4.2 - 5.6)

*Age group*			
Less than 25 years	1008	43	4.3 (3.1 - 5.7)
25 to 30 years	1194	49	4.1 (3.0 - 5.4)
31 to 35 years	613	32	5.2 (3.6 - 7.3)
36 to 40 years	443	29	6.5 (4.4 - 9.3)
41 to 45 years	310	19	6.1 (3.7 - 9.4)
46 and more	325	17	5.2 (3.5 - 8.9)

*Marital status*			

Single	782	34	4.3 (3.0 - 6.0)
Divorced	252	28	11.1 (7.5 - 15.6)
Married-monogamous	2095	83	3.9 (3.2 - 4.9)
Married-polygamous	764	44	5.8 (4.2 - 7.6)

*Study period*			

2006	832	56	6.7 (5.1 - 8.6)
2007	148	16	10.8 (6.3 - 16.9)
2008	433	28	6.5 (4.3 - 9.2)
2009	994	41	4.1 (2.9 - 5.5)
2010	1075	34	3.2 (2.2 - 4.4)
2011	411	14	3.4 (1.9 - 5.6)

**Table 4:** Association between biological disorders and infection with *Trichomonas vaginalis*.

Parameters	Examined	Number of positive	Frequency (%)
			(95%CI)

*Leukocytes*∗			

Absence	104	03	2.9 (0.6 - 8.2)
Rare	2947	102	3.5 (2.8 - 4.2)
Moderate	388	34	8.8 (6.1 - 12.0)
Many	242	21	8.7 (5.4 - 12.9)
High	212	29	13.7 (9.3 - 19.0)

* Erythrocytes*			

Absence	3332	131	3.9 (3.3 - 4.6)
Rare	169	20	11.8 (7.4 - 17.7)
Moderate	285	25	8.8 (5.7 - 12.7)
Many	82	11	13.4 (6.9 - 22.7)
High	25	02	8.0 (0.9 - 26.0)

*Vaginal flora*			

Type I	1186	24	2.0 (1.3 - 2.9)
Type II	634	20	3.1 (1.9 - 4.8)
Type III	576	33	5.7 (3.9 - 7.9)
Type IV	1497	112	7.5 (6.2 - 8.9)

**Table 5:** Factors associated with *Trichomonas vaginalis* infection among participating women.

	Univariate analysis	Multivariate analysis	
Parameters	Odds ratio	Adjusted Odds ratio	p value
	(95%CI)	(95%CI)	

*Vaginal flora*			
Type I	Reference	Reference	-* *-
Type II	1.5 (0.8 - 2.9)	1.6 (0.9 - 3.0)	0.12
Type III	2.9 (1.7 - 5.0)	2.6 (1.5 - 4.4)	0.001
Type IV	3.9 (2.5 - 6.1)	3.3 (2.1 - 5.3)	0.001

*Erythrocytes*			

Absence	Reference	Reference	-* *-
Presence	2.8 (2.0 - 3.9)	2.8 (1.9 - 3.9)	0.001

*Leucocytes*			

Absence	Reference	Reference	-* *-
Presence	1.7 (0.5 - 5.5)	1.3 (0.4 - 4.2)	0.70

*Marital status*			

Single	Reference	Reference	-* *-
Divorced	2.7 (1.6 - 4.6)	2.1 (1.2 - 3.7)	0.009
Married-monogamous	0.9 (0.6 - 1.4)	0.8 (0.5 - 1.3)	0.46
Married-polygamous	1.3 (0.8 - 2.1)	1.3 (0.8 - 2.1)	0.30

*Age group*			

Less than 25 years	Reference	Reference	-* *-
25 to 30 years	1.0 (0.6 - 1.4)	0.9 (0.6 - 1.4)	0.73
31 to 35 years	1.2 (0.8 - 2.0)	1.1 (0.7 - 1.9)	0.59
36 to 40 years	1.6 (0.9 - 2.5)	1.3 (0.8 - 2.2)	0.27
41 to 45 years	1.5 (0.8 - 2.6)	1.1 (0.6 - 2.1)	0.65
46 and more	1.2 (0.7 - 2.2)	0.7 (0.4 - 1.3)	0.28

*Study period*			

2006	Reference	Reference	--
2007	1.7 (0.9 - 3.0)	1.7 (0.9 - 3.1)	0.08
2008	0.9 (0.6 - 1.5)	0.8 (0.5 - 1.3)	0.42
2009	0.6 (0.4 - 0.9)	0.5 (0.3 - 0.8)	0.003
2010	0.4 (0.3 - 0.7)	0.4 (0.3 - 0.6)	0.001
2011	0.5 (0.3 - 0.9)	0.5 (0.3 - 0.9)	0.03

Hosmer Lemeshow Goodness of fit test: Chi (8 ddl)=7.79; p=0.45

Area under the curve (AUC)=0.71; test for Multicollinearity using variance inflation factor (VIF)=1.41 - Akaike information criterion=1423; Bayesian information criterion (BIC)=1542.

## Data Availability

The data used to support the findings of the study are available from the corresponding author upon request.
